# Small cell neuroendocrine carcinoma of the cervix: From molecular basis to therapeutic advances

**DOI:** 10.1016/j.bj.2023.100633

**Published:** 2023-07-17

**Authors:** Angel Chao, Ren-Chin Wu, Chiao-Yun Lin, Ting-Chang Chang, Chyong-Huey Lai

**Affiliations:** a Department of Obstetrics and Gynecology, Chang Gung Memorial Hospital at Linkou, Taoyuan, Taiwan; b Gynecologic Cancer Research Center, Chang Gung Memorial Hospital at Linkou, Taoyuan, Taiwan; c Department of Pathology, Chang Gung Memorial Hospital at Linkou, Taoyuan, Taiwan; d College of Medicine, Chang Gung University, Taoyuan, Taiwan

**Keywords:** Small cell neuroendocrine carcinoma, Uterine cervix, Immune checkpoint inhibitors, Molecular targets

## Abstract

Small cell neuroendocrine carcinoma of the cervix (SCNECC) is an uncommon but aggressive uterine malignancy, the cause of which is generally associated with human papillomavirus (HPV) infection. A lack of clinical trials and evidence-based treatment guidelines poses therapeutic challenges to this rare tumor. At present, published data remain limited to case series and case reports. While clinical management has traditionally followed those of small cell neuroendocrine (SCNE) lung cancer relying on surgery, chemoradiation, and systemic chemotherapy, the prognosis remains dismal. Immune checkpoint inhibitors (ICIs), such as monoclonal antibodies that target programmed death-1 (PD-1) or programmed death-ligand 1 (PD-L1), atezolizumab and durvalumab have proven effective in extensive-stage SCNE lung cancer. Moreover, pembrolizumab has also proven beneficial effects when added onto chemotherapy in metastatic and recurrent HPV-associated non-SCNE cervical cancer. It holds promise to use ICIs in combination with chemoradiation to improve the clinical outcomes of patients with SCNECC. Future advances in our understanding of SCNECC biology – associated with the study of its genomic and molecular aberrations as well as knowledge from SCNE of lung and other extrapulmonary sites– would be helpful in discovering new molecular targets for drug development. Collaborative efforts and establishment of a SCNECC-specific biobank will be essential to achieve this goal.

## Introduction

High-grade neuroendocrine tumors (NETs) represent a highly heterogeneous group of neoplasms derived from ubiquitous neuroendocrine cells, most commonly arising from the lungs, the gastrointestinal tract, and the pancreas [1]. NETs may occasionally develop outside of their most common primary tumor sites, including the female genital tract. Small cell neuroendocrine carcinoma of the cervix (SCNECC) is an aggressive form of NET that affects the uterine cervix [[2], [3], [4]]. It is rare and accounts for less than 2% of all invasive cervical malignancies. Unfortunately, the prognosis of SCNECC remains poor because of its frequent recurrence and the high metastatic burden at diagnosis [5]. Histopathologically, the tumor is composed of round to spindle-shaped cells with an infiltrative growth pattern [6]. Hyperchromatic nuclei, lymphovascular space invasion (LVSI), and necrotic areas are common findings. Immunohistochemical markers of neuroendocrine differentiation – including chromogranin, CD56, and synaptophysin – are usually, but not invariably, positive [7,8]. In addition to the unfavorable outcomes [9], the rarity of the disease poses major therapeutic challenges due to a shortage of clinical trials and lack of evidence-based treatment guidelines. The clinical experience described in case series and case reports still represents the only available source of information of this disease.

In this narrative review, we present an overview of the pathogenesis of SCNECC and elaborate the clinical management of patients with this rare cervical malignancy. Our discussion further summarizes the present knowledgement and advancement in this field. We further delve into the updates on the tumorgenesis of high-grade NETs, especially focusing on pulmonary and extrapulmonary small cell neuroendocrine (SCNE) neoplasms. The objective is to derive strategies that can be potentially applicable to SCNECC. The following sections aim to highlight the aspects that are most relevant to improving our understanding of SCNECC tumorigenesis, thereby developing innovative and effective treatment.

## Deciphering the pathogenesis of small cell neuroendocrine carcinoma of the cervix

### Role of human papillomavirus infection

The majority of SCNECC can be linked to oncogenic human papillomavirus (HPV) infections, with HPV18 being the predominant genotype [6,[10], [11], [12]]. A comprehensive systematic review and meta-analysis of 403 cases revealed that 85% of SCNECC samples were tested positive for HPV [11]. Specifically, following the integration of the viral genome into infected host cells, the main viral oncoproteins, E6 and E7, sequestered the cellular ubiquitin-proteasome system to promote rapid degradation of the tumor suppressor proteins TP53 (tumor protein p53) and Rb1 (RB transcriptional corepressor 1), respectively [13]. Furthermore, the E6 protein may drive tumorigenesis through its ability to activate the expression of the catalytic subunit of telomerase – an enzyme activity which is crucial for immortalization [13]. In addition to binding to Rb, the E7 protein can promote malignant transformation by driving cell cycle progression and disrupting the epigenetic control of normal cellular lifespan through its ability to bind histone deacetylases [13]. The Cancer Genome Atlas Research Network demonstrated that HPV-associated cervical carcinomas exhibit several significantly mutated genes (SMGs) such as *SHKBP1* (SH3KBP1 binding protein 1), *ERRB3*, *CASP8* (caspase 8), *HLA-A* (major histocompatibility complex, class I, A), and *TGFR2* (transforming growth factor beta receptor 2). These SMGs also include newly identified ones like the mutations driven by the apolipoprotein B mRNA editing catalytic polypeptide-like driven mutations, however, no SCNECC was included [14].

### Molecular pathogenesis and prognosis

Knowledge of the molecular pathogenesis of SCNECC is not only crucial for comprehending the malignant transformation process but also instrumental in paving ways towards specific therapies targeting the underlying biological aberrations. Common mutations identified in SCNECC varied across different studies. Xing et al. using targeted next generation sequencing (NGS) of 637 genes, immunohistochemistry and *in situ* hybridization found *TP53, PIK3CA* (phosphatidylinositol-4,5-bisphosphate 3-kinase catalytic subunit alpha)*, KRAS* (KRAS proto-oncogene, GTPase)*, ERBB2* (erb-b2 receptor tyrosine kinase 2)*, c-Myc* (MYC proto-oncogene; bHLH transcription factor)*, Notch1, BCL6* (BCL6 transcription repressor)*, NCOA3* (nuclear receptor coactivator 3)*, PTEN* (phosphatase and tensin homolog)*, RB1, BRCA1/2* (BRCA1/2 DNA repair associated protein)*, and ARID1B* somatic mutations in 4 of 10 SCNECCs [15]. Interestingly, recent comparative genomics data suggested that SCNECC may be genetically closer to common cervical cancer subtypes compared with extra-cervical SCNEs of the lung and bladder [16]. Hillman et al. performed whole exome sequencing of 15 high-grade NECC and found PI3K-kinase or MAPK pathway activating mutations in 67%, including *PIK3CA* p. E545K (n = 4, 27%), *KRAS* p. G12D, *GNAS* p. R201C (n = 2, 13%), and *TP53* (13%) [16]. This suggests that oncogenic *KRAS* activation, aberrant PI3K signaling, and constitutive activation of adenylate cyclase may be part of the molecular alterations associated with SCNECC. Mutations in *PIK3CA* (18%, 11%)*, KRAS* (14%, 11%)*, TP53* (11%, 13%) have been described in different studies using targeted NGS (50 genes and 520 genes), respectively [17,18]. Besides, mutations in p53 pathway were associated with poor prognosis [18].

Using whole exome sequencing followed by functional analysis, Cho et al. [19] found that mutations in *ATRX* (ATRX chromatin remodeler) – a tumor suppressor gene – and *ERBB4* (erb-b2 receptor tyrosine kinase 2) – which encodes a receptor tyrosine kinase in the epidermal growth factor family – can promote SCNECC tumorigenesis. A large cohort consisted of 47 SCNECC tumors were explored and revealed 21% of the tumors were immunoreactive with neurotrophic receptor tyrosine kinase (NTRK) but none of these had *NTRK1-3* gene fusion, while DLL3 (delta-like canonical notch ligand 3) expression could be observed in 81% of the tumors [20]. Subsequently, Chen et al. corroborated the lack of *NTRK1-3* gene fusion in another cohort of 43 patients with SCNECC, with 51.16% testing positive for programmed death-ligand 1 (PD-L1) [21]. DLL3 is frequently expressed in SCNE lung carcinomas but not detectable in normal adult tissues [22], however, a recently published clinical trial using antibody–drug conjugate targeting DLL3 has failed [22]. Genomic landscape in high-grade NECC (n = 97) including 79 SCNECCs found that the most frequently altered genes were *PIK3CA* (19.6%)*, MYC* (15.5%)*, TP53* (15.5%), and PTEN (11.3%) [23]. The genomic landscapes were also quite different between HPV-positive and HPV-negative SCNECCs [23] (Table 1). Furthermore, a large study discovered insulinoma-associated protein 1 (INSM1) was more sensitive (100% positive) than chromogranin (85%) in SCNE lung carcinoma [24]. Kuji et al. studied INSM1 in high-grade NECC and found that INSM1 expression has sensitivity, specificity, positive predictive value, and negative predictive value of 94%, 98%, 88%, and 99%, respecively [25]. In a separate study, they also found that 72% of NECC were HPV-positive [26].

**Table 1:** Comparison of genetic features of high-grade NECC, more common cervical carcinomas, and SCNE lung carcinomas.

Genetic alterations	High-grade NECC^a^ (n = 97) [23]				SCC, AD, ASC [14]	SCNE lung carcinoma (n = 1800) [14]	*p* value	
	HPV+ (n = 83)	HPV- (n = 14)	High-grade NECC (n = 97)	SCNECC (n = 79)			High-grade NECC HPV + *versus* HPV- [23]	SCNECC *versus* SCNE lung carcinoma [23]
*PIK3CA*	17%	36%	19.6%	24.0%	26%	5.1%	0.0037	0.0001
*MYC*	17%	7%	15.5%	12.7%	0.6%	6.3%	0.0484	0.0001
*TP53*	11%	43%	15.5%	12.7%	6%	90.1%	0.0001	0.0001
*PTEN*	8%	50%	14.4%	13.9%	8%	8.9%	0.0001	NS
*ARID1A*	5%	36%	9.3%	10.1%	7%	4.2%	0.0001	0.012
*RB1*	4%	36%	8.2%	6.3%	4%	70.9%	0.0001	0.0001
*KRAS*	NA	NA	8.2%	NA	6%	NA	NA	NA
*BRCA2*	NA	NA	6.3%	NA	3%	NA	NA	NA
MSI-high	NA	NA	2.7%	3.1%	NA	0.004%	NA	0.0001
TMB 6–19 mutations/Mb	NA	NA	16.5%	15.2%	10%	62.3%	NA	0.0001
TMB ≧ 20 mutations/Mb	NA	NA	2.1%	2.5%	4%	8.2%	0.2691	0.0767

Abbreviations: NECC: neuroendocrine cervical carcinoma; HPV: human papillomavirus; SCNECC: small cell neuroendocrine cervical carcinoma; SCC: squamous cell carcinoma; AD: adenocarcinoma; ASC: adenosquamous carcinoma; SCNE: small cell neuroendocrine; PIK3CA: phosphatidylinositol-4,5-bisphosphate 3-kinase catalytic subunit alpha; MYC: MYC proto-oncogene; bHLH transcription factor; TP53: tumor protein p53; PTEN: phosphatase and tensin homolog; ARID1A: AT-rich interaction domain 1 A; RB1: RB transcriptional corepressor 1; KRAS: KRAS proto-oncogene, GTPase; MSI: microsatellite instability; TMB: tumor mutation burden; NA: not applicable; NS: not significant.

a High-grade NECC included 79 SCNECCs and 18 large cell NECC.

## Prognostic factors, current therapeutic protocols, and clinical outcomes

### Surgery or chemoradiation?

Advanced stage, lymph node metastasis, deep stromal invasion, not receiving chemotherapy, and large tumor size are related to prognosis [[27], [28], [29], [30], [31], [32], [33]]. However, clinical outcomes for SCNECC patients tend to vary based on the treatment approach. The challenge of treating patients with SCNECC is featured by the lack of information from randomized clinical trials to guide the selection of optimal therapies [27]. According to the National Comprehensive Cancer Network guidelines, patients with stage I (≤4 cm) SCNECC can be treated with radical hysterectomy and chemotherapy or chemoradiation [28]. However, treatment guidelines for advanced and/or recurrent disease are lacking, and data from a small number of retrospective studies remain the only source to inform patient management [[29], [30], [31], [32], [33]]. At present, the mainstay of treatment includes surgery, radiotherapy, and systemic chemotherapy (Table 2). A study conducted by the Taiwanese Gynecologic Oncology Group (TGOG) [29] enrolled 179 patients with SCNECC. Of the 144 with early-stage disease, 110 underwent primary surgery and 34 received primary radiotherapy. Patients in the latter group (6%) showed a significant reduced rate (*p* = 0.009) of loco-regional failure compared with the former (27%), suggesting that radiotherapy with chemotherapy may yield superior outcomes in terms of loco-regional control. Moreover, in a specific subset of 13 patients with a tumor size <2 cm and no LVSI underwent radical surgery, their 5-year overall survival (OS) was as high as 89% (2 of the 4 without adjuvant chemotherapy recurred and died of disease) [29]. Excluding the 13 patients with excellent prognosis, the 5-year OS rate was significantly better in the 14 patients underwent at least 5 cycles of platinum-based chemothrapy and primary radiotherapy. The OS rate for this group was significantly higher (78%) compared to the 46% in the 97patients (*p* = 0.046) [29]. For advanced disease, the TGOG study analysed patients with stages IIB−IVB. The results showed that chemoradiation with platinum and etoposide (PE), if completed at least five cycles significantly improved both the 5-year failure-free survival (62.5% *versus* 13.1%, respectively; *p* = 0.025) and cancer-specific survival (CSS) (75.0% *versus* 16.9%, respectively; *p* = 0.016) [31]. Unfortunately, the 5-year CSS of 0% was observed for patients with stage IVB [31].

**Table 2:** Summary of published studies (sample size: >50 cases) on treatment approaches and clinical outcomes of patients with small cell neuroendocrine carcinoma of the cervix.

Authors	Number of patients	Disease stage	Treatment modality	Outcomes
Chen et al. [29]	111	I−II	S (n = 97) *vs* RT-EP5+ (n = 14)	5-year OS: 46% *vs* 78%
Cohen et al. [30]	135	I−IIA	S *vs* non-S	5-year OS: 38.2% *vs* 23.8%
	45	IIB−IVA	CT *vs* non-CT	17.8% *vs* 12.0%
	8	IVB		0%
Wang et al. [31]	146	IA-IIB	Treatment comprising surgery *vs* other treatments	5-year FFS 41.2% vs 60.5%, *p* = 0.086
	56	IIB−IVB	CCRT-EP5+ (n = 16) *vs* other treatments	IIB−IVB 5-year CSS: 75% *vs* 16%
		IVB		IVB 0%
Intaraphet et al. [32]	82	I−IIA	S ± CT/RT/CCRT (n = 70), CT only (n = 2), RT only (n = 26), CCRT (n = 32)	5-year CSS: 65.1% (I), 45% (IIA), 25.4% (IIB), 0% (III and IV)
	48	IIB−IV	RT (±CT)	
Lee et al. [33]	102	IA−IV	S (n = 7), NACT + S ± CT/RT/CCRT (n = 55), S + CT (n = 8), S + RT (n = 11), CT only (n = 7), RT only (n = 2), CCRT (n = 24)	Recurrent rate: 51.6%, median TTP: early stage 22.3 months vs advanced 13.3 months (*p* = 0.104)
Yin et al. [34]	64	IB−IVB	S (n = 52), CT (n = 57), RT (n = 40), NACT (n = 22, CCRT (n = 19)	5-year OS: 54.4% (IB−IIA), 9.8% (IIB−IVB)

Abbreviations: *CCRT-EP5+*: *concurrent radiotherapy and at least 5 cycles of etoposide and* platinum chemotherapy; CSS: cancer-specific survival; FFS: failure-free survival; CT: chemotherapy; EP5+: at least five cycles of *etoposide and platinum*; NACT: neoadjuvant chemotherapy; OS: overall survival; Pt: patient; RT: r*adiotherapy;* S: surgery; vs: versus*; TTP*: *time to progression*.

On analyzing a pooled analysis of 188 patients with stages I−IVB SCNECC, Cohen et al. [30] conducted a pool analysis of 188 patients with stage I-IVB SCNECC. They found that the 5-year OS of patients who received radical hysterectomy (38.2%) was higher than those of women who did not receive the operation (23.8%) (*p* < 0.001). This reflects that pateints treated with surgery were at a lower disease or had smaller tumors. In addition, Cohen et al. [30] reported the 5-year CSS rates of 9.8% and 0% in stage IIB−IVA and IVB SCNECC, respectively. Importantly, patients with stage IIB−IVA disease who received chemotherapy (as primary adjuvant or concurrent with radiotherapy) showed a better 3-year OS rate of 17.8% as opposed to the 12% in those who did not receive chemotherapy (*p* = 0.04).

A Thai study highlighted that early-stage SCNECC patients who underwent primary surgery and adjuvant chemotherapy had better survival than those who did not receive adjuvant therapy or adjuvant radiotherapy/chemoradiation [32]. Furthermore, the authors reported that older age (>60 year old) and deep stromal invasion were significant poor prognostic factors [32]. A Korean multi-center study (n = 102) found that parametrial involvement and LVSI were associated with unfavorable prognosis [33]. A study from China found that stage and tumor size were significant independent prognostic factors for early stage SCNECC [34]. Additional study substantiates the finding that postoperative chemotherapy can improve the OS of patients diagnosed with early-stage SCNECC, compared to those who undergo primary surgery alone [35]. Postsurgical chemotherapy regimens have been compared, and the combinations of PE, as well as vincristine, doxorubicin, and cyclophosphamide (VAC), outperforms other treatment regimens [36,37]. Collectively, these observations suggest that tumor size and deep stromal invasion may improve the prognostic stratification of SCNECC in early-stage disease. Moreover, PE chemotherapy has demonstrated an improvement in OS for patients undergoing primary surgery or radiotherapy for stage I-IV disease.

### Role of neoadjuvant chemotherapy

Data on the potential utility of neoadjuvant chemotherapy (NACT) in SCNECC remain inconclusive [38,39]. A study by Dongol et al. [38] described four women with stage IB1−IIB SCNECC who received NACT followed by radical hysterectomy. Unfortunately, only one patient was alive when the study was published. In a Japanese multi-center study of 126 SCNECC patients, it was found that fewer than 4 chemotherapy cycles, paraaortic lymph node metastasis, locally advanced disease and distant metastasis were all associated with a worse outcome [40]. Furthermore, the response rate to PE therapy was found to be superior compared to the taxane-platinum regimen (43.8% vs 12.9%) [40]. Conversely, Lee et al. [39] indicated a worse prognosis for women with stage IB-IIA SCNECC who underwent NACT followed by radical surgery. This study also suggested that the burden of residual diseases following NACT may have a prognostic significance. Accordingly, Bermudez et al. [41] showed that the 5-year OS of women with residual tumors smaller than 2 cm was significantly higher than for those with tumors larger than 2 cm (58% *versus* 21%, respectively; *p* < 0.05). Wang et al. noted a trend towards worse failure-free survival (41.2% vs 60.5%, *p* = 0.086) in patients who received NACT before surgery as compared to those received primary surgery with or without adjuvant therapy [31].

In summary, a wide range of therapeutic regimens have been attempted in the treatment of SCNECC, resulting in a multimodality approach [28]. Generally, patients with early-stage (stage IB1) tumors are recommended to undergo radical hysterectomy followed by adjuvant chemotherapy or chemoradiation. However, primary radical hysterectomy can be reserved for patients whose tumors are less than 2 cm without deep stromal invasion/LVSI, as they can achieve excellent outcome with just postoperative adjuvant chemotherapy, thereby avoiding the need for adjuvant chemoradiation. For patients with more advanced disease, definitive chemoradiation could be an option. As far as chemotherapy is concerned, PE should be currently considered as the most effective regimen. Nonetheless, the role of NACT in SCNECC remains unclear.

## Potential utility of immunotherapy

Recently, ICIs have demonstrated significant improvements in progression-free survival (PFS) in various solid tumors [[42], [43], [44]]. Programmed death-1 (PD-1), a negative co-receptor found in antigen-stimulated B and T cells, has a crucial role in mediating cytotoxic effects on cancer cells [45]. Pembrolizumab (Keytruda®) – a potent humanized immunoglobulin G4 monoclonal antibody – has high binding affinity to the PD-1 receptor and inhibits its interaction with both PD-L1 and programmed cell death ligand 2 (PD-L2). Pembrolizumab has an acceptable preclinical safety profile and is currently as an intravenous immunotherapy for advanced malignancies.

Research has found that HPV-associated cervical cancer expressed increased levels of PD-L1 [46], specifically, HPV16 E7 has the ability to directly induce PD-L1 expression [47]. This has led to the testing of pembrolizumab as a potential treatment for patients with cervical cancer [48]. The phase Ib KEYNOTE-028 trial demonstrated a 17% overall response rate for PD-L1-positive cervical cancer patients [49]. Furthermore, the KEYNOTE-158 trial indicated that pembrolizumab monotherapy produced a durable antitumor activity with manageable safety profile in patients with advanced cervical cancer [49]. The comprehensive evaluation from the KEYNOTE-826 trial, which involve 617 patients with recurrent, persistent, or metastatic cervical cancer. The trial reported that pembrolizumab significantly outperformed placebo plus platinum-based chemotherapy in terms of PFS, having 10.4 months *versus* 8.2 months, respectively (hazard ratio [HR] = 0.65; 95% confidence interval = 0.53−0.79; *p* < 0.001) [42] (Table 3). While the three trials mentioned did not encompass patients with SCNECC, the role of HPV infection in the pathogenesis of this rare malignancy has prompted additional clinical testing of pembrolizumab. The potential of incorporating ICIs into chemoradiotherapy as a front-line treatment for locally advanced cervical cancer is currently being explored [50,51]. Regrettably, a phase II trial on the use of pembrolizumab monotherapy in recurrent SCNECC was discontinued due to an unsatisfactory response [52,53]. Nonetheless, a patient with stage IVB SCNECC (with bone metastasis and liver metastasis) achieved long-term survival benefits from combined pembrolizumab and chemoradiotherapy [54]. Another PD-1 inhibitor, nivolumab, demonstrated therapeutic effect in a patient with large-cell neuroendocrine cervical cancer when coupled with radiotherapy [55]. A patient with metastatic SCNECC who showed a complete response following treatment with nivolumab and radiotherapy [56]. However, despite these promising outcomes, we believe that targeting PD-1/PD-L1 – particularly utilizing ICIs monotherapy – may not suffice to ensure an adequate control of SCNECC after failure of first-line treatment.

**Table 3:** Overview of clinical trials using immune checkpoint inhibitors in recurrent/advanced cervical cancer.

Reference	Number of patients/inclusion criteria	Treatment	Outcomes
KEYNOTE-028 Trial Frenel et al. [49] Phase Ib	37/PD-L1+	Pembrolizumab 10 mg/kg Q2W for 24 m	ORR: 17% PR: 17% SD: 13%
KEYNOTE-158 study Chung et al. [53] Phase II	98/recurrent	Pembrolizumab 200 mg Q3W for 24 m	ORR: 12.2% PR: 9.2% SD: 18.4%
KEYNOTE-826 trial Colombo et al. [48] Phase III	617/persistent/recurrent metastatic	Pembrolizumab 200 mg Q3W plus platinum-based chemotherapy followed by maintenance Pembrolizumab for 35 cycles *vs* placebo Q3W plus platinum-based chemotherapy followed by maintenance placebo for 35 cycles	OS at 24 m: 50.4% *vs* 40.4% *p* < 0.001
KEYNOTE-A18/ENGOT-CX11 Lorusso et al. [50] Phase III	980/untreated stage I/II with 2 PLNs+ or PALN + or stage III/IVA	Five cycles of pembrolizumab 200 mg Q3W + CRT with single agent cisplatin followed by 15 cycles of pembrolizumab 400 mg Q6W *vs* Five cycles of placebo Q3W + CRT with single agent cisplatin followed by 15 cycles of placebo	Currently ongoing
ATOMICC trial [51] Phase II	132 IB2-IVA with PLN or PALN + after CRT	Dostarlimab 500 mg Q3W for 4 doses followed by 1000 mg Q6W up to 24 months *vs* no treatment (2:1 ratio)	Currently ongoing

Abbreviations: CRT: chemoradiotherapy; m: months; ORR: overall response rate; PALN: paraaortic lymph node; PLN: pelvic lymph node; PR: partial response; SD: stable disease; vs: versus; W: weeks.

Notwithstanding, the potential utility of pembrolizumab as an add-on to curative chemoradiation warrants further exploration. For example, data from the KEYNOTE-604 study highlighted that the addition of pembrolizumab to EP as first-line therapy improved both PFS (HR = 0.75, *p* = 0.0023) and OS in patients with extensive SCLC (HR = 0.8, *p* = 0.0164) [57]. Anti-PD-L1, atezolizumab and durvalumab, have also proven efficacy in extensive-stage SCLC as front-line therapy (atezolizumab: HR of OS 0.70, *p* = 0.007 and durvalumab HR of OS 0.73, *p* = 0.0047) [58,59]. These findings suggest that the addition of ICIs to current chemotherapy regimens could enhance therapeutic effects against SCNECC. More studies specifically focusing on SCNECC are necessary to conclusively determine the potential benefits of ICIs.

## Molecular subtypes and targeted therapies in SCNE carcinoma of lung and other extrapulmonary sites

Recent studies have discovered molecular subtypes of SCNE lung cancer defined by *ASCL1* (achaete-scute family bHLH transcription factor 1), *NEUROD1* (neurogenic differentiation 1), *POU2F3* (POU class 2 homeobox 3), and *YAP1* (Yes1 associated transcriptional regulator) transcription factors [60,61]. Targeted agents are actively tested in SCLC and a range of solid tumors (Table 4) [60,61]. Lurbinectedin, an inhibitor of DNA active transcription of protein-coding genes showed high overall response rate (35.2%) in SCNE lung cancer [62], is currently undergoing a randomized trial [63]. Promoters of ASCL1-and NEUROD1-dependent genes were found as specific targets of lurbinectedin in SCNE lung cancer cells [64].

**Table 4:** Overview of clinical trials on targeted therapies in small cell neuroendocrine carcinoma of the lung or other anatomical sites.

ClinicalTrials.gov identifier	Targeted agent	Inclusion criteria/Treatment/Phase
NCT02566993 [63]	DNA transcription inhibitor, Lurbinectedin	Small-cell lung cancer failed one prior line platinum-based chemotherapy/Lurbinectedin (PM01183)/Doxorubicin *versus* CAV or Topotecan (ATLANTIS)/Phase III
NCT02487095 [65]	ATR inhibitor, berzosertib (VX-970)	Metastatic or unresectable malignancies (including 5 SCNE lung cancer, 1 SCNECC/Topotecan with VX-970 (M6620)/Phase I/II
NCT03958045	PARP inhibitor, rucaparib ICI, nivolumab	Platinum-sensitive extensive-stage SCNE lung cancer patients/Combined rucaparib with nivolumab/Phase II
NCT03009682	PARP inhibitor, olaparib	Relapsed SCNE lung cancer patients with HR pathway gene mutations not limited to BRCA 1/2 mutations, ATM deficiency or MRE11A mutations (SUKSES-B)/olaparib/Phase II, single arm
NCT03428607	PARP inhibitor, olaparib ATR kinase inhibitor, ceralasertib (AZD6738)	Relapsed SCNE lung cancer patients (SUKSES-N2)/ceralasertib and olaparib combination therapy/Phase II, single-arm
NCT02937818	PARP inhibitor, olaparib ATR kinase inhibitor, ceralasertib Wee1 inhibitor, adavosertib (AZD1775) ICI, durvalumab and tremelimumab	Platinum refractory extensive-stage SCNE lung cancer/Phase II open-label, multi-arm
NCT02593019 [66] NCT03106155 [66] NCT03366675 [66]	Wee1 inhibitor, adavosertib (AZD1775) mTORC1/2 inhibitor, vistusertib (AZD2014) Aurora kinase B inhibitor, AZD2811	SCNE lung cancer failing platinum-based chemotherapy/*MYC* family amplification or co-alteration in *CDKN2A* and *TP53*(SUKSES-C) or no specific biomarkers (SUKSES-N1) allocated to adavosertib/Phase II SCNE lung cancer failing platinum-based chemotherapy/RICTORamp allocated to vistusertib (SUKSES-D)/Phase II SCNE lung cancer failing platinum-based chemotherapy/no specific biomarkers allocated to AZD2811(SUKSES-N3)/Phase II

Abbreviations: ATM: ataxia telangiectasia mutated; ATR: ataxia-telangiectasia–mutated and rad3-related protein kinase; CAV: cyclophosphamide, doxorubicin, and vincristine; HR: homologous recombination; ICI: immune checkpoint inhibitor; PARP: Poly (adenosine diphosphate–ribose) polymerase; SCNE: small cell neuroendocrine; MYC: MYC proto-oncogene; bHLH transcription factor; TP53: tumor protein p53.

Agents targeting a homologous recombination deficiency, poly (ADP-ribose) polymerase (PARP) inhibitors such as olaparib and rucaparib, as well as ATR kinase inhibitor and Wee1 inhibitors are currently under investigation for their potential in treating SCNE lung cancer (Table 4). Combination of topoisomerase 1 inhibitor, VX-970 (M6620), depleted ataxia-telangiectasia–mutated and rad3-related protein kinase, may stop cancer cells from preventing the repair of DNA damaged by chemotherapy [65]. Among 21 patients with metastatic or unresectable malignancies (comprising five cases of SCNE lung cancer and one case of SCNECC), who were treated with an ATR inhibitor (M6620), three out of the five patients with platinum-refractory SCNE lung cancer had a partial response or prolonged stable disease, lasting 10, ≧ 6, and ≤7 months, respectively [65]. However, the phase III trial which was investigating the use of VX-970 was unfortunately suspended (NCT03896503). Meanwhile, the results from an umbrella trial for recurrent SCNE lung cancer were reported [66]. Patients with *MYC* family amplification or co-alteration in *CDKN2A* and *TP53* were allocated to the SUKSES-C arm (n = 7), while patients lacking specific biomarkers were assigned to the SUKSES-N1 arm (n = 24) and receiving adavosertib. The SUKSES-N3 arm (n = 15) received AZD2811, whereas the SUKSES-D arm (n = 4) were treated with vistusertib. Patients allocated to SUKSES-C & N1 showed no objective response rate, with three (42.9%) and six (25.0%) patients, respectively, achieving stable disease. The median PFS was 1.28 months (95% CI 1.18–not available) and 1.21 months (95%CI 1.15–2.33), respectively. Five patients in the N3 arm (n = 15) had stable disease [66].

Interestingly, Schlafen 11(SLFN11), a protein that acts parallel to homologous recombination pathway, is a biomarker to guide therapy selection in PARP inhibitors [67]. Patients who test SLFN11 positive may be eligible for atezolizumab with or without talazoparib (NCT04334941). Furthermore, EZH2 depletion has been shown to potentiate MYC degradation inhibiting tumor growth in xenograft of small cell carcinoma [68]. EZH2 inhibitor (DS-3201 b), an epigenetic modifier to reverse SLFN11 promoter methylation, is being investigated for recurrent SCNE lung cancer (NCT03879798).

## Conclusions and future directions

The high metastastic potential of SCNECC still poses formidable challenges. Previous therapeutic approaches to this malignancy showed suboptimal efficacy. Our present knowledge on the mechanisms of SCNECC tumorigenesis remains limited and the low quality of evidence has hampered the development of recommendations for clinical management. For now, incorporation of PE chemotherapy in the management of SCNECC of all stages is recommended. Furthermore, ICIs such as anti-PD1/PD-L1, in combination with PE chemotherapy, have proven efficacy in extensive-stage SCNE lung cancer as front-line therapy and recurrent, persistent, or metastatic cervical cancer. Thus, SCNECC patients not in the best prognostic group (<2 cm, no LVSI/deep stromal invasion) may be offered add-on ICIs out of clinical trials. Several shortcomings of published studies in the field should be considered, including 1) small sample sizes due to the rarity of SCNECC, 2) lack of detailed information on its molecular underpinnings, and 3) the inherent biases of non-randomized study designs. In the future, two main research directions await investigation. First, new therapeutic approaches to SCNECC may be informed by positive results obtained in other NETs, and may thus receive specific scrutiny in SCNECC. Second, a comprehensive dissection of the underlying molecular aberrations, including genetic and epigenetic drivers, will be a key prerequisite to enhance therapeutic outcomes. When coupled with collaborative biobanking, which is paramount to achieve sufficiently large samples in the field of rare malignancies, it is anticipated that high-throughput, integrative “omics” approaches supported by advanced bioinformatics tools will permit to uncover exhaustive biological information on SCNECC that will offer substantial support for future tailored treatments.

## Conflicts of interest

The authors declare that they have no known competing financial interests or personal relationships that could have appeared to influence the work reported in this paper.
